# Spatial Analysis and Correlates of County-Level Diabetes Prevalence, 2009–2010

**DOI:** 10.5888/pcd12.140404

**Published:** 2015-01-22

**Authors:** J. Aaron Hipp, Nishesh Chalise

**Affiliations:** Author Affiliation: Nishesh Chalise, Brown School, Washington University in St. Louis, St. Louis, Missouri.

## Abstract

**Introduction:**

Information on the relationship between diabetes prevalence and built environment attributes could allow public health programs to better target populations at risk for diabetes. This study sought to determine the spatial prevalence of diabetes in the United States and how this distribution is associated with the geography of common diabetes correlates.

**Methods:**

Data from the Centers for Disease Control and Prevention and the US Census Bureau were integrated to perform geographically weighted regression at the county level on the following variables: percentage nonwhite population, percentage Hispanic population, education level, percentage unemployed, percentage living below the federal poverty level, population density, percentage obese, percentage physically inactive, percentage population that cycles or walks to work, and percentage neighborhood food deserts.

**Results:**

We found significant spatial clustering of county-level diabetes prevalence in the United States; however, diabetes prevalence was inconsistently correlated with significant predictors. Percentage living below the federal poverty level and percentage nonwhite population were associated with diabetes in some regions. The percentage of population cycling or walking to work was the only significant built environment–related variable correlated with diabetes, and this association varied in magnitude across the nation.

**Conclusion:**

Sociodemographic and built environment–related variables correlated with diabetes prevalence in some regions of the United States. The variation in magnitude and direction of these relationships highlights the need to understand local context in the prevention and maintenance of diabetes. Geographically weighted regression shows promise for public health research in detecting variations in associations between health behaviors, outcomes, and predictors across geographic space.

## Introduction

More than 25 million Americans have diabetes, and another 80 million have prediabetes; taken together, approximately 1 in 3 Americans have diabetes or prediabetes (1). Diabetes is associated with obesity and physical inactivity; many built environment factors — attributes of the proximate environment — such as access to healthy foods (2), crime level (3), the rural–urban matrix (4–6), and walking (4) are correlated with diabetes prevalence. One of the great challenges in understanding the associations between built environment attributes and diabetes is that both factors vary across the United States. Although studies of diabetes have found spatial variations in incidence and prevalence, there is a paucity of information on how the spatial prevalence of diabetes may or may not be associated with the spatial prevalence of built environment attributes. The importance of understanding the covariance of diabetes with its correlates was made salient by Siordia and colleagues (7), who found that the relationship between poverty and diabetes prevalence varied across the United States, with poverty highly correlated with diabetes in some regions but not in others (7). This finding provided the impetus for our hypothesis that the relationship between diabetes prevalence and county-level built environment attributes is nonstationary (ie, the relationship varies across space).

The objective of our study was to determine how and where diabetes prevalence is associated with built environment attributes at the county level in the contiguous United States. This information could allow programs and interventions to better target populations and attributes of the built environment associated with high diabetes prevalence.

## Methods

Our study used geographically weighted regression (GWR), a tool that is increasingly used by public health researchers to understand the nuances of such issues as access to health care, disease distribution, and spatial variation in magnitude of health outcome predictors (8–10).

### Data sources

We used county-level cross-sectional secondary data from various publicly available sources. Geographic information systems (GIS) shapefiles of contiguous US counties were downloaded from the Topographically Integrated Geographic Encoding and Referencing (TIGER) files available from the US Census Bureau (11) and imported into the ArcGIS 10.2 software (ESRI). Data on diabetes prevalence, obesity rates, and physical inactivity were collected from the Centers for Disease Control and Prevention’s (CDC’s) Diabetes Interactive Atlas (12), which is based on data from the Behavioral Risk Factor Surveillance System (BRFSS). CDC defines diabetes prevalence as the estimated percentage of adults with diagnosed diabetes, after adjusting for age. BRFSS does not differentiate between type 1 and type 2 diabetes. CDC defines obesity prevalence as the estimated percentage of obese adults (body mass index ≥30) after adjusting for age. The prevalence of physical inactivity is an estimated percentage of adults who are physically inactive. Physically inactive adults are those who have not participated in any physical activity or exercise in the preceding 30 days (http://www.cdc.gov/diabetes/library/glossary.html). All BRFSS data are based on self-report. Data on walking or cycling to work were collected from the US Census; this variable was defined as the percentage of employed adults per county who stated they either walked or cycled to work in the previous week.

Data for the sociodemographic variables — percentage nonwhite population, percentage Hispanic population, percentage living below the federal poverty level, education level, population density, and percentage unemployed — were from the US Census Bureau’s American Community Survey 5-year estimates (2006–2010) (13). The variable for percentage nonwhite population refers to the percentage of people who did not identify themselves as white and does not include Hispanics who identify themselves as white. Percentage Hispanic population refers to the percentage of people who identified themselves as Hispanic (both white and nonwhite). The percentage of people living below the federal poverty level was determined according to income thresholds defined by the US Census Bureau, which differ by family composition. The education variable was defined as the percentage of people who reported having less than a high school diploma. Population density was defined as the number of people per square mile in a county. Unemployment was determined as the percentage of civilians aged 16 years or older that did not have work for the reference week. Data on food deserts were collected from the Department of Agriculture (USDA); the food desert variable was defined as the percentage of census tracts (per county) that are food deserts (http://www.ers.usda.gov/data-products/food-access-research-atlas/download-the-data.aspx). USDA defines a census tract as a food desert if 33% of the population lives far (urban, >1 mile; rural, >10 miles) from a supermarket or a grocery store. All variables were determined at the county level. There were 3,109 counties included in the study. Counties in Alaska and Hawaii were excluded because we could not test the influence of proximity; these states do not border other US states, and in Hawaii, no county borders another.

### Geographically weighted regression

We used GWR in addition to ordinary least squares (OLS) regression because the spatial data used in our study violates 2 major assumptions of global regression. First, global OLS regression assumes observations are independent of each other. However, spatial data often are clustered, suggesting stronger relationships between proximate observations (14). Clustering can result in correlation among regression residuals across space, or spatial autocorrelation, and biased parameter estimates (15). Second, OLS regression assumes spatial stationarity of the relationship between independent and dependent variables (16). In other words, it assumes coefficients will be constant across a sample area. However, the context of a particular area can influence the magnitude and direction of the relationship and produce a range of coefficients (17). GWR relaxes these assumptions and enables the analysis of spatially relevant data. Unlike OLS regression models, which produce global models across space, GWR produces numerous local models. It simultaneously conducts multiple regressions so that there is one regression model per spatial data point (eg, a county). Observations closer to a particular data point will have more weight in the estimation than observations farther away.

### Methodological steps in model building

The first step in the model building process is to map the dependent variable and explore spatial heterogeneity. If the dependent variable is not clustered, there is no need to build a spatially explicit model. Without clustering, the global model will be similar to the local model (17). We used the Moran’s Index (*I*) in ArcGIS to map the clustering of diabetes prevalence across counties in the United States. Moran’s *I* ranges from −1.0, perfectly dispersed (eg, a checkerboard pattern), to a +1.0, perfectly clustered. A *z* score and *P* value are generated as outputs along with Moran’s *I*.

Initial data exploration and model specification using OLS was completed using SPSS 22 software (IBM Corporation). Three factors motivated the decision to first specify the OLS model: 1) we wished to identify variables significantly correlated with the dependent variable before specifying the regression model; 2) the GWR software used for spatial analysis does not provide a variance inflation factor (VIF) to assess multicollinearity; and 3) the GWR software does not enable the researcher to extract regression residuals to assess spatial autocorrelation for the global model.

In the OLS regression we included only variables significantly correlated with the dependent variable, diabetes prevalence. Residuals from the global OLS model were mapped and analyzed for spatial autocorrelation using Moran’s *I*. The same set of variables was then used to specify a GWR model using the GWR4 software (http://geodacenter.asu.edu/gwr). While conducting GWR, we used the adaptive kernel, which was produced using the bi-square weighting function. The adaptive kernel uses varying spatial areas but a fixed number of observations for each estimation, a method most appropriate when the distribution of observations varies across space. In our case, observations (counties) are much smaller and closer together in the Northeast and Southeast than they are in the Midwest and West Coast. Finally, a process that minimizes the Akaike Information Criteria (AIC) was used to determine the best kernel size. The parameter estimates and *t* values produced by the software were exported and mapped using ArcGIS 10.2 (ESRI).

The residuals of GWR models are assumed to be normally distributed; a further assumption is that they are not spatially autocorrelated or clustered across space. Such clustering suggests that the local model underestimates or overestimates diabetes prevalence in particular areas. The residuals from the GWR model were analyzed using Moran’s *I* to assess spatial autocorrelation. The clustering of residuals for OLS and GWR models were compared to assess the value of using GWR.

### Comparison of OLS and GWR model performance

We used 3 tools to compare the OLS and GWR models. First, we compared the adjusted R^2^ of the basic OLS model and the GWR model. A higher adjusted R^2^ in the GWR model than in the OLS model for the same set of variables suggests that location plays an important role in explaining the variance of diabetes prevalence. Second, we compared the corrected AIC (AICc) for both models. AICc is a widely used measure of goodness-of-fit that adjusts for degrees of freedom (18). It can be used to compare models with the same dependent variable but different independent variables. AICc can also be used to compare a global model with local models (17) because AICc does not assume models must be nested (18). The values of AICc are not absolute, but relative, so that they are meaningful only when compared between models. The model with a smaller AICc is deemed a better fit. The final analytical step was to compare residuals of both models for their distribution and spatial autocorrelation.

## Results

Diabetes prevalence in the United States at the county level ranged from 3.8% to 17.8% and was significantly clustered (Moran’s *I* = 0.35; *z* = 540.2; *P* < .001). We found clusters of high diabetes prevalence in the Southeast and clusters of low diabetes prevalence in Colorado. Diabetes prevalence was significantly correlated with numerous independent variables. Because the percentage of neighborhood food deserts was not significantly correlated at the county level, it was not included in the OLS model. The following 9 variables were included in the OLS model: population density, percentage nonwhite, percentage Hispanic, percentage living below the federal poverty level, percentage with less than high school education, percentage unemployed, percentage obese, percentage physically inactive, and percentage that walked or cycled to work. The OLS model was significant (*F*_9,3099_ = 495.87, *P* < .001). The model explained 58.8% of the variance in county-level diabetes prevalence. The VIF for all variables was less than 4.0, a commonly used cutoff point, suggesting no multicollinearity (Table 1).

**Table 1:** Results From Ordinary Least Square Model of US County-Level Diabetes Prevalence, 2009–2010

Characteristic	β	SE	*t* Value	*P* Value	Variance Inflation Factor
Intercept	4.80	0.190	24.94	<.001	—
Population density	0.000192	0	8.93	<.001	1.45
Percentage nonwhite population	0.043	0.002	26.22	<.001	1.42
Percentage Hispanic population	−0.03	0.002	−18.32	<.001	1.11
Percentage living below federal poverty level	0.10	0.004	23.65	<.001	1.46
Percentage unemployed	−0.89	0.360	−2.48	.01	2.95
Percentage with less than a high school education	0.44	0.110	3.97	<.001	3.00
Percentage obese	0.063	0.009	6.99	<.001	2.22
Percentage physically inactive	0.03	0.007	5.86	<.001	2.15
Percentage that walks or cycles to work	−12.46	0.580	−21.38	<.001	1.47

Abbreviation: SE, standard error.

The residuals of the OLS model were spatially autocorrelated (Moran’s *I* = 0.13; z = 26.4; *P* < .001). The OLS model overestimated diabetes prevalence for Colorado and New Mexico counties. Similarly, it underestimated the outcomes for Alabama and West Virginia counties.

**Figure 1:**
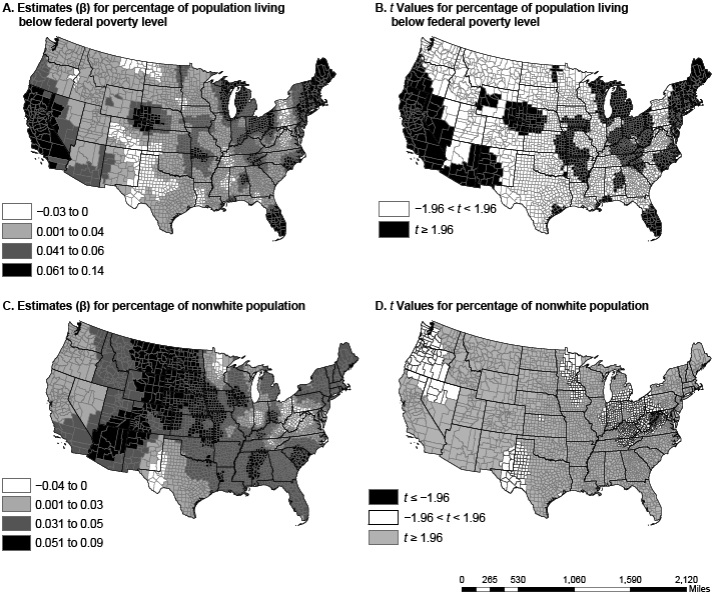
Spatial variation in parameter estimates and *t* values in US counties for the percentage of people living below the federal poverty level (maps A and B) and the percentage of nonwhite population (maps C and D). Data sources: American Community Survey (2006–2010) (13) and Centers for Disease Control and Prevention (12).

**Figure 2:**
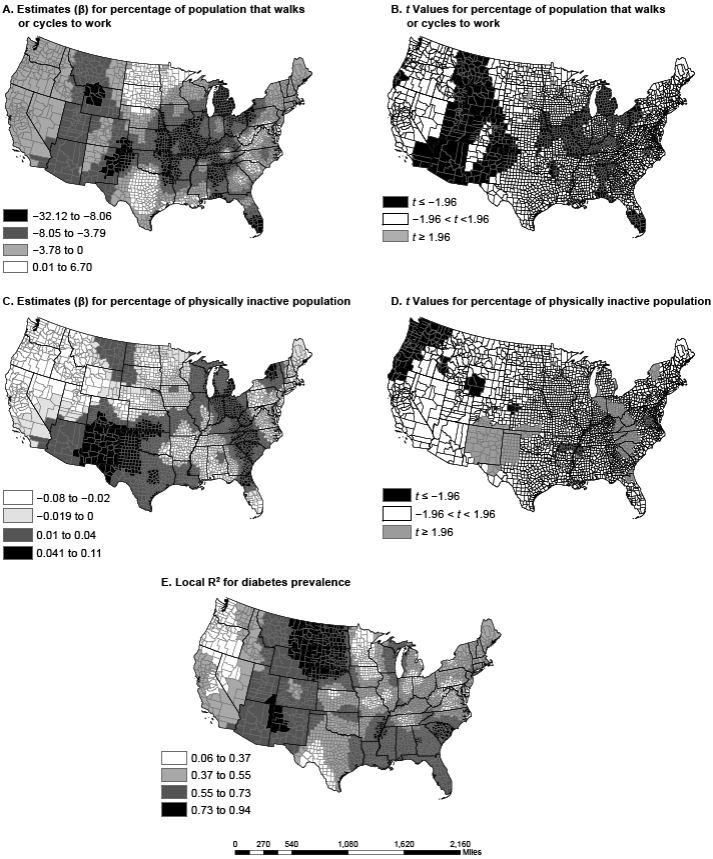
Spatial variation in parameter estimates and *t* values in US counties for percentage of employed population walking or cycling to work (maps A and B) and the percentage of the population that is physically inactive (maps C and D); local R-squared for full geographically weighted regression model (Map E). Data sources: American Community Survey (2006–2010) (13) and Centers for Disease Control and Prevention (12).

The GWR model produced coefficients for each county (Table 2, Figure 1, Figure 2). The change in both magnitude and direction of the coefficients suggests spatial nonstationarity of the relationship between the predictors and diabetes prevalence. The direction of the relationship in most counties was as expected. Only a few counties had opposite relationships for the predictors in the GWR model. In most counties, walking or cycling to work was associated with lower diabetes prevalence. However, a few clusters of rural counties in Minnesota, North Dakota, and South Dakota show an association between walking or cycling to work and higher diabetes prevalence. Such nonstationarity demands a more nuanced analysis with a contextual focus. For example, high rates of walking or cycling to work are often associated with multimodal transportation that also includes public transit, which is less likely to be available in rural communities (19).

**Table 2:** Results From Geographically Weighted Regression Model of US County-Level Diabetes Prevalence, 2009–2010

Characteristic	β		Percentage of Counties by 95% of *t* Statistic		
	Min	Max	*t *≤ −1.96	−1.96 < *t* < 1.96	*t* ≥ 1.96
Intercept	1.60	10.7	0	0	100
Population density	−0.003	0.01	13.2	86.2	0.70
Percentage nonwhite population	−0.04	0.09	1.00	21.6	77.4
Percentage Hispanic population	−0.22	0.16	30.4	68.0	1.50
Percentage living below federal poverty level	−0.02	0.14	0.00	60.2	39.8
Percentage unemployed	−45.4	23.4	21.4	78.1	0.60
Percentage with less than a high school education	−6.66	15.0	0.10	76.3	23.6
Percentage obese	−0.07	0.16	0.00	71.0	29.0
Percentage physically inactive	−0.08	0.11	5.00	81.9	13.1
Percentage that walks or cycles to work	−32.1	6.69	29.2	70.7	0.20

**Table:** 

The figure consists of 4 maps of the contiguous US states with estimates and t values of the variables poverty and nonwhite based on a geographically weighted regression (GWR) for county-level diabetes prevalence. Map A shows the estimates of poverty ranging from −0.03 to 0.14. The variable represents the percentage of population living below the federal poverty level in a county. Based on map B with t values for the estimates of poverty, the negative effect sizes are not significant. Of the positive estimates, 40% are significant. The greatest effect, with significance, of poverty on diabetes is in the Southwest and Southern California, southern part of Florida, and in the Northeast.
Map C displays the GWR estimates of nonwhite population ranging from −0.04 to 0.09. This variable represents the percentage of population that identifies as nonwhite in a county regressed on diabetes prevalence. Based on map D, 78% of the county estimates are significant. The greatest effect sizes for the nonwhite population on diabetes prevalence are in the Dakotas south through Nebraska and into northern Kansas and Missouri.

**Table:** 

The figure consists of 5 maps of the 48 contiguous US states. Maps A through D have estimates and t values for the variables walking or biking to work and physical inactivity based on a geographically weighted regression (GWR) for county-level diabetes prevalence. Map A shows the estimates of the variable walking or biking to work ranging from −32.12 to 6.70 per county. This variable represents the percentage of people in a county who walked or biked to work in the preceding week. Only 30% of the estimates are significant, which is shown in map B with t values. There are 2 main regions where walking or biking to work has a significant inverse relationship with diabetes prevalence (higher rate of walking or biking to work is associated with lower diabetes prevalence). One region follows the spine of the Rocky Mountains. The second region is in the Midwest and the Mississippi and Ohio River valleys. There are 5 counties where walking and biking has a significant positive relationship with diabetes prevalence, each in South Dakota.
Map C shows estimates for physical inactivity ranging from −0.08 to 0.11. This variable represents the percentage of people in a county who did not participate in physical activity or exercise in the preceding 30 days, regressed on diabetes prevalence. Only 18% of the county estimates for this variable are significant based on the t values in map D. Physical inactivity has a significant inverse relationship with diabetes in the Northwest. It has significant positive relationship (higher rates of physical inactivity associated with higher rates of diabetes) in New Mexico and parts of Texas.
Map E displays the local R-squared values based on the GWR for diabetes prevalence. Local R-squared values range from 0.06 to 0.94. Local R-squared represents the amount of variance in diabetes prevalence explained by the model in each county. The model performed the best in parts of the country stretching from the Dakotas and Montana south to New Mexico and Arizona, as well as in the Southeast. The GWR model was not able to explain much variance in the Northwest, parts of the upper Midwest, and the Mid-Atlantic.

The adjusted R^2^ for the local GWR model ranged from 0.06 to 0.94; the adjusted R^2^ in the OLS model was 0.58. Explicitly, the global OLS R^2^ of 0.58 masks a wide distribution of local associations between the predictors and diabetes prevalence. Without GWR, we would have been unable to estimate local models. In counties in North Dakota, South Dakota, and Montana, the GWR model explained up to 94% of the variance in diabetes prevalence. However, in Washington and Oregon, the model did not explain much of the variance (6%–37%), a spatial variation that would have been missed with the OLS model alone. Residuals for the GWR model, although significant, were less spatially autocorrelated than residuals for the OLS model (Moran’s *I* = 0.01; *z* = 3.74; *P* < .001). Compared with OLS, the GWR model greatly improved model fit. The GWR model explained more variance in diabetes prevalence and reduced the AICc (ΔR^2^ = 0.22; ΔAICc = 2,008.4).

## Discussion

Poverty level, physical inactivity, and walking or cycling to work were each significantly associated with county-level diabetes prevalence, relationships that were spatially nonstationary across the United States. The variation in parameter estimates from GWR suggests the need to apply this spatial analysis tool to other diabetes studies that have been restricted to global models (2,4). In the global OLS model, 58.8% of county-level diabetes prevalence was explained by race, poverty, obesity, physical inactivity, and walking or cycling to work. However, at an individual county level, the explanatory percentage ranged from 6% to 94%, and the individual county-level models were significantly clustered. This clustering suggests that local contexts, policies, programs, and built environment attributes are associated with diabetes prevalence and that the amplitude of such contexts, policies, programs, and environments varies across the nation.

The dissimilarity in variable coefficients was not a factor of one county alone but was a factor of multiple proximal counties, perhaps because of policy and programmatic spillover from neighboring counties and diffusion of innovation (20). The percentage of nonwhite population in a county had the greatest effect in the Southeast and the Rockies, from Arizona and New Mexico to Idaho and Montana. States in these regions have a high proportion of African Americans, Hispanics, or Native Americans, races/ethnicities with disproportionately high rates of diabetes (21). In several regions (including the Midwest, the Ohio Valley, and New England), poverty had a greater association with diabetes prevalence than any other variable. Physical inactivity had the greatest effect in the Southeast and the Southwest, a pattern similar to that of obesity prevalence (22). Walking or cycling to work was most associated with diabetes prevalence in the Mississippi Valley, the panhandles of Texas and Oklahoma, and south Florida, areas not generally associated with walking or cycling because of their hot summers.

The relationships among nonwhite populations, poverty, physical inactivity, and diabetes are not new (3,4,7). Others found these relationships have a spatial component (23). With the exception of recent work by Siordia and colleagues (7), there has been no investigation into the nonstationarity of these relationships. Similarly, the strong association between walking or cycling to work and diabetes is consistent with findings of other studies (24), but it has not been investigated for spatial heterogeneity or nonstationarity. That there is a significant association between nonwhite populations, poverty, physical inactivity, and diabetes and that this relationship has a spatial but nonstationary association highlights the need for local, context-specific diabetes prevention programs.

There are limitations to GWR and our analyses. GWR equates the local regression coefficients based on those geographic areas (eg, counties) most proximate to the area of interest. That is, the regression equation and coefficients for a county in Missouri are most influenced by bordering counties and other nearby counties, but not influenced by counties in Colorado or North Carolina. This concept is essential for local planning and related to Tobler’s first law of geography, that “everything is related to everything else, but near things are more related than distant things” (25). However, the distance of influence (of predictors or potential interventions) is theoretically unknown and perhaps inconsistent across a geographic area (eg, the continental United States). We chose to use an adaptive kernel bandwidth, which accounted for differences in the size of counties and therefore the distance of influence. This choice should have helped adjust for the fact that, for example, North Carolina has 100 small counties and California has 58 larger counties spread over 3 times the landmass of North Carolina. Because of this discrepancy, the data point (county) was an estimate based on proximate counties as defined by the kernel type. GWR is also limited by the edge effect, whereby counties located on the edges of the United States (ie, coastal regions and the borders with Canada and Mexico) do not have the 360° influence of counties in the nation’s interior.

There are also limitations to our findings. The local R^2^s accounted for 6% to 94% of county-level diabetes prevalence. In large geographic areas in the Mid-Atlantic, upper Midwest, and Northwest, the 9 variables included in the model explained less than one-third of the variance in diabetes prevalence, which means that most factors associated with county-level diabetes prevalence in these geographic areas must have been missing from our model.

The primary strength of this study is the use of GWR in the analysis of the spatial distribution and correlates of diabetes prevalence. Siordia and colleagues (7) introduced the concept of spatial nonstationarity to the relationship between poverty and diabetes. Here, we extend their work by incorporating additional socioeconomic variables and built environment correlates with diabetes. GWR adds value to public health research and practice by emphasizing location-specific theories of health outcomes and tailored policies for intervention. It scrutinizes the assumption of global relationships between various predictors and health outcomes. Using GWR, public health researchers and practitioners can gain a nuanced understanding of health-related issues and respond to the notion that “all health is local” (25,26). In doing so, they can provide clarity for designing and funding context-specific public health programs and policies, especially for national programs that have local reach, including those of the CDC and the American Diabetes Association (ADA). Our analyses could also be used by local public health departments and ADA offices for resources such as MIYO (Make It Your own - http://www.miyoworks.org/) to tailor messages and materials for their target audiences. The use of GWR is a key advancement in public health research and practice because many health behaviors and outcomes vary spatially (eg, obesity) as do many common predictors (eg, race/ethnicity) (27).

Shedding light on spatial variations can provide new insights into well-established relationships. The methodology of GWR needs to be expanded to additional public health efforts to understand the impact of environment and place on health and how these relationships may vary across space. For diabetes prevalence, we presented an initial step in this direction, but much work remains before we understand why these variations exist and why race/ethnicity, poverty, physical inactivity, and active commuting have little explanatory effect in some regions but explain up to 94% of diabetes prevalence in other regions.
